# Impact of smoking and smoking cessation on overweight and obesity: Scotland-wide, cross-sectional study on 40,036 participants

**DOI:** 10.1186/1471-2458-13-348

**Published:** 2013-04-15

**Authors:** Daniel F Mackay, Linsay Gray, Jill P Pell

**Affiliations:** 1 Institute of Health and Wellbeing, University of Glasgow, 1 Lilybank Gardens, Glasgow, G12 8RZ, UK; 2 MRC Social and Public Health Sciences Unit, University of Glasgow, 4 Lilybank Gardens, Glasgow, G12 8RZ, UK

**Keywords:** Body mass index, Obesity, Overweight, Smoking, Smoking cessation

## Abstract

**Background:**

Weight control is cited by some people, especially adolescent girls, as a reason for commencing smoking or not quitting. The aim of this study was to explore the relationship between smoking behaviour and being overweight or obese, overall and by age and sex sub-groups.

**Methods:**

We used data from the six Scottish Health Surveys conducted to date (1995–2010) to undertake a population-based, cross-sectional study on 40,036 participants representative of the adult (≥16 years) Scottish population. Height and weight were measured by a trained interviewer, not self-reported.

**Results:**

24,459 (63.3%) participants were overweight (BMI ≥25 kg/m^2^) and 9,818 (25.4%) were obese (BMI ≥30 kg/m^2^). Overall, current smokers were less likely to be overweight than never smokers. However, those who had smoked for more than 20 years (adjusted OR 1.54, 95% CI 1.41-1.69, p < 0.001) and ex-smokers (adjusted OR 1.18, 95% CI 1.11-1.25, p < 0.001) were more likely to be overweight. There were significant interactions with age. Participants 16–24 years of age, were no more likely to be overweight if they were current (adjusted OR 1.01, 95% CI 0.84-1.20, p = 0.944) or ex (adjusted OR 0.88, 95% CI 0.67-1.14, p = 0.319) smokers. The same patterns pertained to obesity.

**Conclusions:**

Whilst active smoking may be associated with reduced risk of being overweight among some older adults, there was no evidence to support the belief among young people that smoking protects them from weight gain. Making this point in educational campaigns targeted at young people may help to discourage them from starting to smoke.

## Background

Published evidence suggests an association between smoking behaviour and weight [1-3]. Overall, weight is lower among those who smoke and higher among those who have quit. However, previous studies suggest that the relationship may be more complex. A causal association between smoking and lower weight is plausible, but evidence also exists for reverse causation. Those who are overweight and attempting to lose weight are more likely to start smoking [4,5]. Some studies suggest that the apparent protective effect of smoking in relation to obesity may, in fact, have been restricted to long-term and light smokers [6]. Among younger smokers, there may be no association between smoking and weight and, among older smokers, heavy smoking may be associated with higher weight [6-8].

Fear of gaining weight is sometimes cited as a reason for starting or not quitting smoking. The factors that influence smoking behaviour vary by age and sex [5]. Perceived weight, body weight concerns and dieting behaviours are stronger drivers of smoking behaviour in adolescents; in particular adolescent girls [4,5]. Most adult smokers started smoking during their adolescence [9]. In line with most developed countries, the overall prevalence of smoking in Scotland has fallen over time [10-12]. However, temporal trends have varied by age and sex. The dramatic decline in smoking prevalence observed in the older age-groups, has been less marked among adolescents and young adults [13,14]. Historically, women had a lower prevalence than men, but the decline in prevalence has been much greater in men [10,11]. Therefore, smoking prevalence is now comparable in both sexes. For these reasons, the smoking behaviour of women and young people will be an important driver of the future overall prevalence of smoking and smoking related conditions. The aim of this study was to examine the relationship between active smoking and smoking cessation and overweight and obesity, overall and by age and sex sub-group, and to explore the effects of smoking duration and dose, and time from cessation.

## Methods

### Scottish health surveys

Scotland has a population of around 5.3 million. The Scottish Health Surveys are periodic cross-sectional surveys of the Scottish general population and are used to evaluate the health and health care needs of the population [10]. An interviewer-administered questionnaire is used to determine self-assessed physical and mental health, illness and disability, lifestyle risk factors (such as smoking, alcohol, and physical activity), health service use and medication and the trained interviewer measures height and weight according to standard operating procedures. The Survey was first conducted in 1995, repeated twice over the next 12 years and became annual from 2008 onwards. The Survey attempts to recruit different people to each survey rather than conducting serial measurements on the same individuals. The Survey data are openly available to researchers and can be downloaded online (http://www.scotland.gov.uk/Topics/Statistics/Browse/Health/scottish-health-survey).

### Inclusion and exclusion criteria

We used data from all six of the surveys available to date (1995, 1998, 2003, 2008, 2009, 2010). These comprised a total of 51,750 adult (≥16 years of age) participants; ranging from 6,465 to 9,017 per individual survey. We excluded participants with a body mass index (BMI) consistent with moderate to severe thinness because some of these individuals may have had anorexia or illnesses that cause cachexia.

### Definitions

BMI is calculated from weight and height: BMI = weight (kg) / (height (m))^2^. Among those aged ≥18 years, moderate to severe thinness was defined as a BMI <17 kg/m^2^, obese as a BMI ≥30 kg/m^2^ and overweight as a BMI ≥25 kg/m^2^. In the comparison of overweight versus normal weight, overweight included obese individuals. For individuals aged 16 and 17 years of age, we used the amended cut-offs recommended by Cole et al. which effectively equated to a 1.0 kg/m^2^ lower BMI among those aged 16 years and 0.5 kg/m^2^ lower BMI among those aged 17 years [15]. Smoking status was defined as current, ex or never smoker based on self-classification by participants. For current smokers, the self-reported number of cigarettes smoked each day was categorized into 1–9, 10–19 and ≥20. For ex-smokers, the time since quitting was categorized into <1, 1, 2–4, 5–9, 10–19 and ≥20 years. Number of quit attempts ever undertaken was classified into none, one/two and three or more. Age at participation was categorized into 16–24, 25–44, 45–64 and ≥65 years. Diabetes was based on a self-reported, physician diagnosis. For alcohol use, participants were classified into never drinkers, ex-drinkers, and nine categories of drinker defined by the number of units consumed weekly: <1, 1–7, 8–10, 11–14, 15–21, 22–28, 29–35, 36–50 and >50. The presence of a mental health problem was based on self-report. Postcode of residence was used to allocate participants to a socioeconomic quintile of the general population using the 2004 Scottish Index of Multiple Deprivation (SIMD) [13]. The index is derived from 31 area markers of deprivation relating to health, education, housing, current income, employment access and crime, that are applied to each postcode data zone. There are 6,505 data zones in Scotland with a mean population of 750 [16].

### Statistical analyses

Logistic regression analyses were used to test the overall association between smoking status and being overweight. Regression models were then applied to the sub-group of current smokers to examine the association between both the number of cigarettes smoked per day and smoking duration and overweight. In the sub-group of ex or current smokers, we examined the association with time from cessation using current smokers (time from cessation = 0) as the referent group. In the sub-group of ex-smokers, we examined whether there was an association with number of quit attempts and, whether it was independent of time from cessation. All associations were tested using univariate and multivariate analyses. The potential confounders included as covariates in the latter analyses were: age, sex and SIMD quintile, diabetes, alcohol use and mental health. In all regression analyses, we tested whether there were statistically significant interactions with age and sex. Where these existed, the models were re-run, stratified by age and sex sub-group. All of these analyses were repeated using obesity as the outcome of interest.

## Results

Of the 44,334 adult participants in the surveys, 154 were excluded from the study because their BMI was consistent with moderate to severe thinness and a further 4,144 because BMI was not recorded. Of the remaining 40,036, complete data on smoking status and BMI were available on 38,668 (96.6%). Of these: 11,475 (29.6%) were current smokers, 10,650 (27.5%) ex-smokers and 16,543 (42.8%) never smokers; 24, 459 (63.3%) were overweight and 9,818 (25.4%) were obese; 3,806 (9.8%) were aged 16–24 years, 13,924 (36.0%) 25 to 44 years, 14,004 (36.2%) 45 to 64 years and 6,934 (17.9%) 65 years or older; 17,257 (44.6%) were male and 21,411 (55.4%) were female. Complete data on the other potential confounders (SIMD quintile, diabetes, alcohol use and mental health) were available on 34,003 and these participants were included in the logistic regression models.

### Smoking status

**Figure 1:**
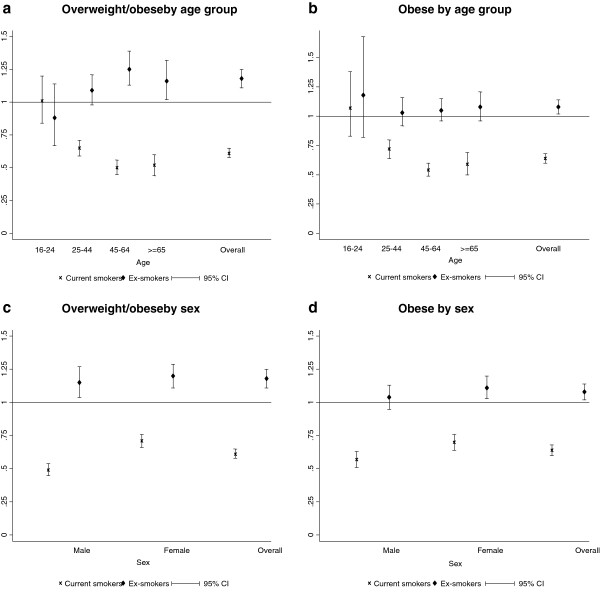
Forest plots of adjusted* odds ratios for the association between smoking status and overweight/obese^†^ and obese^‡^ by age group and sex.

10,615 (64.2%) of the never smokers were overweight, compared with 6,184 (52.7%) current smokers (p < 0.001) and 7,660 (70.3%) ex-smokers (p < 0.001) (Table 1). In the logistic regression analysis, adjustment for potential confounders attenuated some of the association between smoking status and being overweight (Figure 1). Nonetheless the associations remained statistically significant. In comparison with never smokers, current smokers were at reduced risk of being overweight (adjusted OR 0.61, 95% CI 0.58-0.65, p < 0.001) and ex smokers were at increased risk (adjusted OR 1.18, 95% CI 1.11-1.25, p < 0.001). However, there were statistically significant interactions with both age group (P < 0.001) and sex (P < 0.001). Among 16 to 24 year olds, the percentage of never smokers who were overweight (33.7%) was not significantly different from current smokers (36.0%, p = 0.173) and ex smokers (32.9%, p = 0.762) (Table 1). In this age-group, on adjustment for potential confounders in the multivariate model, there remained no statistically significant association with current smoking (adjusted OR 1.01, 95% CI 0.84-1.20, p = 0.944) and smoking cessation (adjusted OR 0.88, 95% CI 0.67-1.14, p = 0.319) and being overweight (Figure 1).

**Table 1:** Prevalence of overweight/obesity and obesity according to smoking status

		**Overweight**			**Obesity**		
		**never**	**current**	**ex**	**never**	**current**	**ex**
		**N = 16,543**	**N = 11,475**	**N = 10,650**	**N = 16,543**	**N = 11,475**	**N = 10,650**
		**N (%)**	**N (%)**	**N (%)**	**N (%)**	**N (%)**	**N (%)**
Overall		10,615 (64.2)	6,184 (53.9)	7,660 (71.9)	4,356 (26.3)	2,271 (19.8)	3,191 (30.0)
Age (years)	16-24	678 (33.7)	473 (36.0)	158 (32.9)	220 (10.9)	161 (12.3)	55 (11.5)
	25-44	3,727 (60.0)	2,423 (50.4)	1,799 (61.9)	1,411 (22.7)	878 (18.3)	679 (23.4)
	45-64	4,126 (75.1)	2,605 (61.6)	3,366 (78.7)	1,802 (32.8)	979 (23.1)	1,442 (33.7)
	≥ 65	2,084 (73.9)	683 (60.5)	2,337 (78.3)	923 (32.7)	253 (22.4)	1,015 (34.0)
Sex	Male	4,810 (69.7)	2,869 (55.5)	4,002 (77.2)	1,768 (25.6)	915 (17.7)	1,524 (29.4)
	Female	5,805 (60.2)	3,315 (52.6)	3,658 (66.9)	2,588 (26.8)	1,356 (21.5)	1,667 (30.5)

*N* number.

Overall, 4,366 (25.9%) never smokers were obese, compared with 2,274 (19.8%) current smokers (p < 0.001) and 3,193 (29.4%) ex-smokers (p < 0.001) (Table 1). In the logistic regression analysis, adjustment for potential confounders attenuated some of the association between smoking status and being obese (Figure 1). Nonetheless, the associations remained statistically significant. In comparison with never smokers, current smokers were at reduced risk of being obese (adjusted OR 0.64, 95% CI 0.60-0.68, p < 0.001) and ex-smokers were at increased risk (adjusted OR 1.08, 95% CI 1.02-1.14, p = 0.014). However, there were statistically significant interactions with both age group (P < 0.001) and sex (p = 0.005). Among 16 to 24 years old, the percentage of never smokers who were obese (10.8%) was not significantly different to current smokers (12.8%, p = 0.078) and ex smokers (12.1%, p = 0.414) (Table 1). On adjustment for potential confounders in the multivariate model, there remained no statistically significant association with current smoking (adjusted OR 1.07, 95% CI 0.83-1.38, p = 0.609) and smoking cessation (adjusted OR 1.18, 95% CI 0.82-1.68, p = 0.374) and being obese in the youngest age group (Figure 1).

### Smoking dose and duration

Among current smokers, there was no clear dose-relationship across the range of number of cigarettes smoked daily. Participants who smoked 10–19 cigarettes per day were not significantly different from those who smoked 1–9 per day in terms of overweight and obesity (Table 2) in either the univariate or multivariate analyses (Table 3). However, those who smoked more than 20 cigarettes per day were significantly more likely to be overweight and obese (Table 2) and adjustment for age, and other potential confounders, did not attenuate the association (Table 3). There was no statistically significant interaction with age in relation to either overweight (p = 0.765) or obesity (p = 0.530). Similarly, the interaction terms were not statistically significant for sex (p = 0.148 and p = 0.148 respectively). In comparison with never smokers, those who smoked more than 20 cigarettes per day were significantly less likely to be either overweight (adjusted OR 0.64, 95% CI 0.59-0.70, p < 0.001) or obese (adjusted OR 0.68, 95% CI 0.62-0.74, p < 0.001) (Additional file 1: Table S1).

**Table 2:** Prevalence of overweight/obesity and obesity by number of cigarettes smoked by current smokers

		**Overweight**			**Obesity**		
		**1-9**	**10-19**	≥**20**	**1-9**	**10-19**	≥**20**
		**N = 2,387**	**N = 4,599**	**N = 4,227**	**N = 2,387**	**N = 4,599**	**N = 4,227**
		**N (%)**	**N (%)**	**N (%)**	**N (%)**	**N (%)**	**N (%)**
Overall		1,231 (51.6)	2,311 (50.3)	2,465 (58.3)	438 (18.3)	796 (17.3)	970 (22.9)
Age (years)	16-24	155 (33.9)	212 (34.5)	102 (46.4)	58 (12.7)	66 (10.7)	37 (16.8)
	25-44	525 (50.7)	937 (47.3)	925 (53.4)	175 (16.9)	336 (17.0)	357 (20.6)
	45-64	390 (62.9)	913 (58.1)	1,200 (63.2)	148 (23.9)	311 (19.8)	474 (24.9)
	≥ 65	161 (58.8)	249 (57.5)	238 (63.5)	57 (20.8)	83 (19.2)	102 (27.2)
Sex	Male	508 (54.6)	980 (51.2)	1,230 (58.2)	147 (15.8)	277 (14.5)	434 (20.5)
	Female	723 (49.7)	1,331 (49.6)	1,235 (58.4)	291 (20.0)	519 (19.3)	536 (25.4)

*N* number.

**Table 3:** Univariate and multivariate logistic regression of the association between the number of cigarettes smoked per day by current smokers and overweight/obesity and obesity

	**Univariate**				Multivariate*				Multivariate**			
	**OR**	**95% CI**	**P value**	**Overall p value**	**OR**	**95% CI**	**P value**	**Overall p value**	**OR**	**95% CI**	**P value**	**Overall p value**
Overweight/Obesity^†^												
1-9	1.00			<0.001	1.00			<0.001	1.00			<0.001
10-19	0.95	(0.86-1.05)	0.295		0.92	(0.83-1.02)	0.096		0.92	(0.82-1.03)	0.139	
≥20	1.31	(1.19-1.45)	<0.001		1.21	(1.09-1.34)	0.001		1.26	(1.12-1.42)	<0.001	
Obesity^‡^												
1-9	1.00			<0001	1.00			<0.001	1.00			<0.001
10-19	0.93	(0.82-1.06)	0.279		0.92	(0.81-1.05)	0.222		0.92	(0.80-1.06)	0.261	
≥20	1.33	(1.17-1.50)	<0.001		1.33	(1.16-1.51)	<0.001		1.31	(1.13-1.50)	<0.001	

*OR* odds ratio, *CI* confidence interval.

*adjusted for age, sex and socioeconomic deprivation quintile.

^**^ adjusted for age, sex, socioeconomic deprivation quintile, diabetes, alcohol and mental health.

^†^compared with normal weight.

^‡^compared with normal or overweight.

Among current smokers, duration of smoking was associated with risk of overweight and obesity even after adjustment for age: adjusted OR for overweight 1.42, 95% CI 0.84-2.00, p < 0.001; adjusted OR for obese 1.43 (95% CI 0.90-1.96, p < 0.001. In comparison with never smokers, those who smoked for more than 20 years were significantly more likely to be either overweight (adjusted OR 1.54, 95% CI 1.41-1.69, p < 0.001) or obese (adjusted OR 1.25, 95% CI 1.15-1.35, p < 0.001).

### Smoking cessation

**Figure 2:**
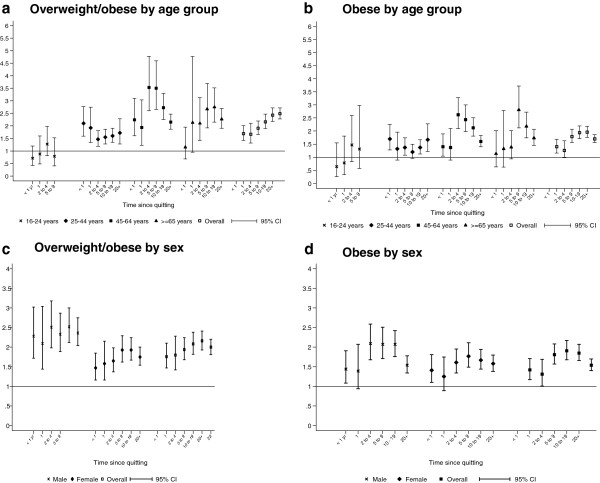
Forest plots of adjusted* odds ratios for the association between time since quitting and overweight/obese^†^ and obese^‡^ among ex-smokers by age group and sex.

Overall, the increase in overweight associated with ceasing smoking occurred largely within one year of smoking (Figure 2). Thereafter, overweight increased more steadily up to 20 years post cessation. Adjustment for age, and other potential confounders attenuated the relationship but there was still some evidence of a weak dose relationship. The interaction with age group was statistically significant (p < 0.001). Among those aged 16–24 years there was no statistically significant association with overweight for either quitting overall or time from cessation (Figure 2a). In contrast, in older age-groups the risk of overweight was increased among all ex-smokers irrespective of time from cessation. The interaction with sex was also statistically significant (both p < 0.001). The effect of smoking cessation on overweight/obesity was less among women irrespective of time from cessation (Figure 2). Among ex smokers, those who were overweight or obese had smoked on average 1.4 years longer than those who were normal weight.

Among ex-smokers, there was no evidence of a significant association between the number of quit attempts and being overweight. In contrast, there was evidence of a dose-relationship with obesity with the risk of obesity being higher among those who had required three or more quit attempts even after adjusting for time from cessation (adjusted OR 1.59, 95% CI 1.22-2.07, p = 0.001) than among those who succeeded after only one or two attempts (fully adjusted OR 1.45, 95% CI 1.11-1.89, p = 0.006).

## Discussion

### Main findings

Overall, current smokers were less likely to be overweight and obese than never smokers. However, people who had smoked for more than 20 years were more likely to be overweight than those who had smoked less and those who had never smoked. People who smoked more than 20 cigarettes per day were more likely to be overweight than those who had smoked less but were still less likely to be overweight than never smokers. The apparent overall protective effect of smoking was not observed among those aged 16–24 years, in whom there was no significant association between smoking status and overweight or obesity. Overall, ex smokers were more likely to be overweight and obese than either never or current smokers. The association was apparent within one year of cessation and increased only slowly over time thereafter. However, there was no evidence of weight gain after cessation among ex-smokers aged 16–24 years, in whom there was no significant difference in either overweight or obesity.

### What is already known

Smoking behaviour is associated with weight [1-3]. Behavioural, sensory and metabolic pathways have all been considered as possible mechanisms by which active smoking may cause reduced weight [17-20]. The evidence for reduced calorific intake, due to smoking instead of eating, impaired smell or taste, or a change in food preference, is largely anecdotal. The evidence for a peripheral metabolic effect is more robust. In vivo animal experiments suggest that administration of nicotine can reduce weight, without a reduction in calorific intake, through less efficient absorption and storage of calories, and an increased metabolic rate and thermogenesis resulting in increased energy expenditure [1,21,22]. Since nicotine is a cholinergic agonist that readily crosses the blood brain barrier a central effect on eating or drinking behaviour is hypothetically plausible but yet to be established. However, the association may also be due, in part, to reverse causation since overweight adolescents with a history of dieting are more likely to start smoking [21]. Smoking can also affect weight distribution, increasing central fat accumulation and hence the risk of metabolic syndrome, diabetes and cardiovascular disease [21,23]. Younger smokers have a shorter period of tobacco consumption and a higher metabolism because they are in a period of natural growth both of which may offset the effects of smoking on body weight observed among older smokers [1].

Some studies have suggested that, whereas light smokers tend to weigh less, heavy smokers tend to weigh more [7,8,21]. This may be more likely among women in socioeconomically deprived communities, where a combination of heavy smoking and severe obesity is more common [24]. A positive causal association between heavy smoking and weight has been suggested on the grounds that heavy smokers are more concerned that smoking cessation may lead to weight gain and are, therefore, less likely to succeed in quitting [25].

### Strengths

The analyses were undertaken on a large, representative sample of the Scottish population. We were able to adjust for potential confounders including area socioeconomic deprivation. Our study covered both sexes and the full age-range enabling us to test for interactions and undertake sub-group analyses. We had sufficient information to examine potential dose relationships in relation to number of cigarettes smoked, smoking duration, time from cessation and number of quit attempts.

### Limitations

Because the study was cross-sectional in nature, it is not possible to establish a temporal relationship between smoking behaviour and weight. Therefore, reverse causation cannot be excluded. Self-reported smoking status was not corroborated using biochemical assays, such as cotinine. Cotinine was only measured in 41% of survey participants who participated in an additional nurse interview and this sub-group may not have been representative of all participants. However, in this sub-group only 3% of those who classified themselves as non-smokers had cotinine levels consistent with current smoking. Due to lack of information on diet, we were unable to explore whether any effect of smoking on weight was mediated via differences in calorific intake. As with any observational study, residual confounding cannot be excluded.

## Conclusions

Temporal trends in smoking prevalence suggest that young adults are particularly resistant to current attempts to reduce smoking. This is, in part, due to their belief that it will adversely affect their weight. This study adds to the evidence that whilst older smokers are less likely to be overweight than non-smokers, this does not appear to be the case for young smokers. Making this point in future educational campaigns aimed at young people may help to discourage them from starting to smoke.

## Competing interests

The authors declare that they have no competing interests.

## Authors’ contributions

JPP conceived the original idea. All authors agreed the study methodology. DFM and LG obtained the data. DFM and LG undertook the statistical analyses. All authors agreed the interpretation of the results. JPP drafted the manuscript. All authors revised the manuscript and approved the final version.

## Pre-publication history

The pre-publication history for this paper can be accessed here:

http://www.biomedcentral.com/1471-2458/13/348/prepub

## Supplementary Material

Additional file 1: Table S1 Univariate and multivariate ordinal logistic regression analysis of the association between smoking dosage and overweight and obese. Click here for file
