# Supplementary material for: Impact of smoking and smoking cessation on overweight and obesity: Scotland-wide, cross-sectional study on 40,036 participants
Source: BMC Public Health. 2013 Apr 15;13:348. doi: 10.1186/1471-2458-13-348 (PMC3636072; doi:10.1186/1471-2458-13-348)
Supplement: Additional file 1: Table S1 — Univariate and multivariate ordinal logistic regression analysis of the association between smoking dosage and overweight and obese. [file 1471-2458-13-348-S1.doc]

**Supplementary Table.** Univariate and multivariate ordinal logistic regression analysis of the association between smoking dosage and overweight and obese

*adjusted for age, sex and socioeconomic deprivation quintile

|  | normal vs  overweight & obese | | | normal & overweight vs  obese | | |
| --- | --- | --- | --- | --- | --- | --- |
|  | OR (95% CI) | P value | P value for trend | OR (95% CI) | P value | P value for trend |
| Univariate |  |  |  |  |  |  |
| 1 -9 | 1.0 |  | <0.001 | 1.0 |  | <0.001 |
| 10-19 | 0.94 (0.86 1.04) | 0.257 |  | 0.93 (0.82, 1.06) | 0.262 |  |
| ≥20 | 1.31 (1.18, 1.45) | <0.001 |  | 1.32 (1.16, 1.50) | <0.001 |  |
|  |  |  |  |  |  |  |
| Multivariate* |  |  |  |  |  |  |
| 1 -9 | 1.0 |  | <0.001 | 1.0 |  | <0.001 |
| 10-19 | 0.91 (0.82, 1.01) | 0.080 |  | 0.91 (0.80, 1.04) | 0.179 |  |
| ≥20 | 1.21 (1.08, 1.34) | 0.001 |  | 1.30 (1.14, 1.48) | <0.001 |  |
|  |  |  |  |  |  |  |
| Multivariate** |  |  |  |  |  |  |
| 1 -9 | 1.0 |  | <0.001 | 1.0 |  | <0.001 |
| 10-19 | 0.92 (0.82, 1.03) | 0.135 |  | 0.91 (0.80, 1.05) | 0.197 |  |
| ≥20 | 1.27 (1.13, 1.42) | <0.001 |  | 1.28 (1.11, 1.47) | 0.001 |  |

^**^ adjusted for age, sex, socioeconomic deprivation quintile, diabetes, alcohol and mental health
